# Experimental Studies on the Impact of the Projected Ocean Acidification on Fish Survival, Health, Growth, and Meat Quality; Black Sea Bream (*Acanthopagrus schlegelii*), Physiological and Histological Studies

**DOI:** 10.3390/ani11113119

**Published:** 2021-10-31

**Authors:** Fabrice Arnaud Tegomo, Zhiwen Zhong, Achille Pandong Njomoue, Samuel Ukpong Okon, Sami Ullah, Neveen Anandi Gray, Kai Chen, Yuxiao Sun, Jinxing Xiao, Lei Wang, Ying Ye, Hui Huang, Qingjun Shao

**Affiliations:** 1 Ocean Academy, Zhejiang University, Zhoushan 316100, China; 11734055@zju.edu.cn (F.A.T.); 21717069@zju.edu.cn (Z.Z.); samukpong@zju.edu.cn (S.U.O.); 11717042@zju.edu.cn (S.U.); 21734181@zju.edu.cn (N.A.G.); 21917027@zju.edu.cn (K.C.); 21817032@zju.edu.cn (Y.S.); xiaojx@zju.edu.cn (J.X.); 11617014@zju.edu.cn (L.W.); gsyeying@zju.edu.cn (Y.Y.); huih@zju.edu.cn (H.H.); 2 Ocean College, Zhejiang University, Zhoushan 316100, China; 3 College of Animal Sciences, Zhejiang University, Hangzhou 310058, China; 4 International College, Zhejiang University, Hangzhou 310058, China; 5 Ecole Nationale Supérieure Polytechnique de Douala-Université de Douala (ENSPD-UD), Logbessou-PK17, Douala 2701, Cameroon; njopanac@enspd-udo.cm; 6 Ocean Research Center of Zhoushan, Zhejiang University, Zhoushan 316100, China

**Keywords:** climate change, growth performance, histology, metabolic acidosis, microvilli atrophy, ocean acidification, seawater pH

## Abstract

**Simple Summary:**

This study’s data suggest that under the projected scenarios of ocean acidification by 2100 and beyond, significant negative impacts on growth, health, and meat quality are expected, particularly on black sea bream, and will be susceptible to the scientifically approved fish having a weaker resistance to diseases and environmental changes if CO_2_ emissions in the atmosphere are not curbed. Knowing the expected consequences, mitigation measures are urgently needed.

**Abstract:**

Acidification (OA), a global threat to the world’s oceans, is projected to significantly grow if CO_2_ continues to be emitted into the atmosphere at high levels. This will result in a slight decrease in pH. Since the latter is a logarithmic scale of acidity, the higher acidic seawater is expected to have a tremendous impact on marine living resources in the long-term. An 8-week laboratory experiment was designed to assess the impact of the projected pH in 2100 and beyond on fish survival, health, growth, and fish meat quality. Two projected scenarios were simulated with the control treatment, in triplicates. The control treatment had a pH of 8.10, corresponding to a pCO_2_ of 321.37 ± 11.48 µatm. The two projected scenarios, named Predict_A and Predict_B, had pH values of 7.80-pCO_2_ = 749.12 ± 27.03 and 7.40-pCO_2_ = 321.37 ± 11.48 µatm, respectively. The experiment was preceded by 2 weeks of acclimation. After the acclimation, 20 juvenile black sea breams (*Acanthopagrus schlegelii*) of 2.72 ± 0.01 g were used per tank. This species has been selected mainly due to its very high resistance to diseases and environmental changes, assuming that a weaker fish resistance will also be susceptibly affected. In all tanks, the fish were fed with the same commercial diet. The seawater’s physicochemical parameters were measured daily. Fish samples were subjected to physiological, histological, and biochemical analyses. Fish growth, feeding efficiency, protein efficiency ratio, and crude protein content were significantly decreased with a lower pH. Scanning electron microscopy revealed multiple atrophies of microvilli throughout the small intestine’s brush border in samples from Predict_A and Predict_B. This significantly reduced nutrient absorption, resulting in significantly lower feed efficiency, lower fish growth, and lower meat quality. As a result of an elevated pCO_2_ in seawater, the fish eat more than normal but grow less than normal. Liver observation showed blood congestion, hemorrhage, necrosis, vacuolation of hepatocytes, and an increased number of Kupffer cells, which characterize liver damage. Transmission electron microscopy revealed an elongated and angular shape of the mitochondrion in the liver cell, with an abundance of peroxisomes, symptomatic of metabolic acidosis.

## 1. Introduction

The full report of the Intergovernmental Panel on Climate Change (IPCC) published in 2019 [1] informed that from the pre-industrial period (1850–1900) until the present time, 2021, the Industrial Revolution involving the anthropogenic emission of CO_2_ into the atmosphere has led to climate change, causing ocean warming and acidification [2,3,4,5,6,7,8,9,10]. Considering the land-based greenhouse gas (GHGs), the fifth assessment report on climate change (AR5) conveyed with medium confidence that the annual net CO_2_ emissions from anthropogenic land-use change were 0.9 (0.1–1.7) gigaton of carbon per year (GtC∙yr^–1^), on average, from 2002 to 2011. From 1750 to 2011, CO_2_ emissions from fossil fuel combustion have released a mean value estimated as 375 (345–405) GtC to the atmosphere, while deforestation and other land-use changes have released an estimated mean value of 180 (100–260) GtC. Of these cumulative anthropogenic CO_2_ emissions, a mean of 240 (230–250) GtC have accumulated in the atmosphere, 155 (125–185) GtC have been taken up by the ocean, and a mean value of 160 (70–250) GtC have accumulated in terrestrial ecosystems [1,11]. Currently, industrialization is exponentially higher than before. As a result, the projected Ocean Acidification (OA) in the coming century is assumed to negatively impact aquatic ecosystems, with increased risks of environmental pollution and a significant threat to its living resources [12,13,14,15,16,17,18].

As a consequence of the industrial revolution, the atmospheric concentration of carbon dioxide (CO_2_) is continually increasing in our oceans [19], thereby increasing the concentration of hydrogen ions in seawater. Seawater consequently shifts into the acidic direction, decreasing the ocean’s environmental pH. According to the IPCC [1], global warming resulting in OA has caused a 0.1 reduction in ocean pH. Remarkably, this slight decrease in the ocean’s pH (i.e., ∆ pH = 0.1) from pre-industrial times until the present corresponds to a 26% to 30% increase in water acidity due to its logarithmic scale, pH =−log [H^+^] (i.e., [H^+^] = 10^(-pH)) [20]. This slight increase of 0.1 in the pH value is significant when considering the pollution and other negative effects on the marine environment and marine species [19,20,21,22,23,24,25,26,27,28,29]. Furthermore, a model projection scenarios indicate that by 2100 and beyond (23rd century), the oceans will warm up by about 3 to 4 °C, leading to an additional decrease of pH estimated from 0.3 to 0.4 in 2100 and 0.7 to 0.8 in the 23rd century, depending on the region, habitat, and emission scenario [13,30,31,32]. It is estimated that the ocean pH in the pre-industrial period was about 8.18. Nowadays, this pH has dropped to around 8.08 [1,25,33]. An important number of articles have reported that such a slight decrease of ∆pH = 0.1, corresponding to 26% increase of acidity had a significant negative impact on the marine environment and species [13,30,31,32]. How much impact will the projected decrease of 0.3 in pH, which is an increase of 99.53% in water acidity, have at the end of the 21st century? And how much impact will a further decrease of 0.7 in pH, which is an increase of about 401.19%, have? We have designed this study to measure the impacts on marine fish and see what mitigating measures are needed.

Considering the great benefit of black sea bream (*Acanthopagrus schlegelii*) to the world fishing economy (6703 tons in 2014) and aquaculture industries (154,389 tons in 2014 including mariculture) [34,35], there is a need to study and understand the risks of OA on marine fish to take preventives measures. The present research intends to evaluate the negative effects of OA on fish survival, health, growth, and fish meat quality. Our targeted values correspond to the projected acidification pH values of 7.80 and 7.40 projected in 2100 and beyond, respectively. It is worth mentioning that Han et al. [3] used these same projected pH values and explained how OA might threaten the population recruitment of broadcast spawning marine species. Likewise, Zhao et al. [36] demonstrated how the pH values 7.80 and 7.40 affected mussels, damaging their shell structure and reducing their shell strength and closure strength. Cao et al. [12] confirmed that due to OA, an elevated pCO_2_ impairs pacific oysters’ immune function, increasing the risk of enhanced disease of marine mollusks. Furthermore, Araujo et al. [20] reported the negative synergistic impacts of ocean warming and acidification on the survival and proteome of gilthead seabream (*Sparus* *aurata*). 

The black sea bream inhabits shallow waters of with 1 to 50 m of depth [37], and has an excellent meat quality and a fast growth rate. This fish species is also known for its efficient feed conversion, high market value, and particularly its high resistance to diseases and environmental changes [38,39,40]. The Data from Nansei National Fisheries Research Institute, published by Sako [41], reported a trend of cultured fish diseases in Japan. They investigated the resistance to diseases of more than thirteen species of fish, including black sea bream. In their results, black sea bream was classified as the most resistant species, having the lowest case (17) of diseases reported, followed by the second more resistant fish, dark-banded rockfish (*Sebastes inermis*), with 21 cases, up to the less resistant one, yellowtail (*Seriola quinqueradiata*), with 1428 cases of diseases [41]. All these characteristics qualify the black sea bream as a potentially essential aquaculture species for offshore culture in the Yellow Sea, the Bohai Sea, the East China Sea, and the South China Sea, also affected by OA [42]. It is assumed that if black sea bream, with a high disease resistance, is affected by OA, other fish species with weaker resistance will be highly susceptible. To the best of our knowledge, no previous studies have considered the effect of the rising OA on the growth and survival of black sea bream, an important aquaculture candidate in Asian countries, as mentioned earlier [34,35]. The present experimental study seeks to understand the implications of the projected 7.80 and 7.40 pH values, respectively, on the survival, growth, health, and meat quality of black sea bream (*Acanthopagrus schlegelii*).

## 2. Materials and Methods

### 2.1. Experimental Design and Procedure, Seawater Parameters

**Figure 1:**
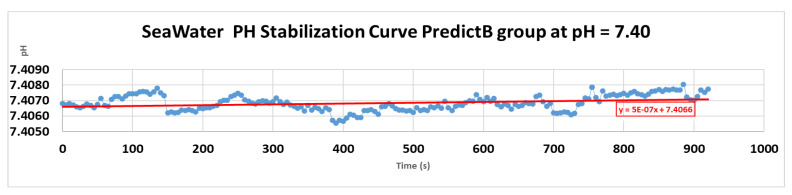
PH stabilization curve of treatment 3, pH = 7.40. The variation of pH is constant at 7.4066 ± 0.02 according to the regression line equation in red.

Three different pH values were used as three treatments, each treatment having three replicates for statistical analysis. The seawater source supply was pumped from the sea, decanted, and well filtered by sand. That seawater supply, with its initial physicochemical characteristics, was directly used as our control group and had a constant pH of 8.10 ± 0.01 during the experiment. This first group corresponds to the present condition of the marine environment. The second and third groups, respectively Predict_A and Predict_B, are the future projection values of pH, respectively pH = 7.80 and pH = 7.40, projected to occur in the year 2100 and beyond. These last two values of pH, 7.80 and 7.40, were obtained by a mixture of dry air and pure CO_2_ and kept stable through low-pressure flow controllers LZM-6T and 100–1000 mL/min oxygen air gas flowmeter with a control valve to adjust CO_2_, working at a pressure *p* = 1atm = 101,325 Pa [3,29,36]. Each tank was filled with 200 L of seawater, with a system of a constant water inflow/outflow of 2 L/min. The pH values in each tank were checked daily by the pH meter Hach SC200 Universal Controller. Before utilization, the pH meter Hach SC200 was calibrated with standard NBS buffers. The pH meter Hach was put inside the seawater tank for 1 h to make sure the evolution of the pH curve over time stayed constant. The constancy of the pH curve over time and the CO_2_ flow controller valve were adjusted only once at the end of the week according to the slight increase of the regression line displayed (Figure 1). The total alkalinity (TA) was assessed through potentiometric titration with an SM-Titrino 702 automatic titrator system [36]. The carbonate chemistry parameters, pCO_2_ (µatm), bicarbonate ions (HCO_3_^−^) (µmol/kg), carbonate ions (CO_3_^2−^) (µmol/kg), dissolved inorganic carbon (DIC) (µmol∙kg^−1^), and the saturation state of aragonite and calcite, respectively Ωara and Ωcal, were all calculated from the measured salinity, measured pH, measured temperature, and measured total alkalinity TA, at an atmospheric pressure of 1atm = 1013.25 kilobar, using the open-source software “CO2cal 1.2.0”. CO2cal 1.2.0 is the updated version of CO2SYS, previously used by many other articles [3,29,36].

#### pH Stabilization Curve

The following picture displays the stabilization of the pH value in treatment 3, in the Predict_B group, where the desired pH value is 7.40. 

For the sake of a high and stable temperature, the experiment was conducted in the summer, from June to August, at the Marine Fisheries Research Institute of Zhejiang Province, Zhoushan City, China, located in Xixuan Island, to take advantage of the high and stable temperature of 27.53 ± 0.85 °C. Black sea bream fingerlings with an initial weight of 1.52 ± 0.03 g were obtained from a private fish farm and transported by boat to the laboratory. Before initiating the main experiment, all fish were first stocked in a single, very large tank, with a capacity of about 14,824 L, and the following dimensions: 4.62 m × 2.63 m × 1.22 m. It was opaque, blue, and made of fiberglass. All fish were fed with a commercial diet (42% crude protein, Ming-Hui Feed Co. Ltd., Zhejiang, China) for 2 weeks of acclimation, to get used to the environmental conditions of Xixuan Island and to overcome the stress caused by the transportation [39,43,44]. Only the surviving and healthy fish remaining after acclimation were selected for the experiment. After the 2 weeks of acclimation, 20 healthy fish, black sea bream fingerlings with an initial weight of 2.72 ± 0.01 g (mean ± SD), were carefully selected and stocked per tank. Each of the three groups had three replicates for a total of nine tanks. Each of the nine smaller tanks had a diameter *Φ* = 80 cm and a height of 65 cm, with a cone-shaped bottom also made of opaque blue fiberglass with a capacity of 350 L. The fish were maintained under a natural photoperiod. The temperature, the ammonia-nitrogen, and the salinity of the seawater in the tanks were, respectively, 27.53 ± 0.85 °C, 0.02–0.04 mg∙L^−1^, and 27.13 ± 1.06 g∙L^−1^. The dissolved oxygen concentration was maintained above the safe value of 5.0 mg∙L^−1^—more precisely, at 7.05 ± 0.51 mg∙L^−1^—at any point during the experiment by continuous aeration with air-stones [39,43,44]. 

For the first two weeks of the experiment, fish were fed three times a day (08:00, 12:00, and 16:00), and then, for the remaining 6 weeks, twice a day (08:00 and 16:00). The fish were hand-fed little by little until apparent satiation, for the efficacy of feeding and to prevent uneaten feed waste. To avoid feed nutrient leaching and to have a very stable feed in the seawater, we used an expanded diet, which means a floating diet that is a professional commercial feed. The fish would swim to the water surface to ingest the feed pellets because the expanded feed floats in water. As long as the fish were fed to apparent satiation, they would not come up to the water surface again. Hence, their apparent satiation could be judged visually [39,43]. The experiment lasted for 56 days (8 weeks), and feed consumption was recorded daily. The tanks were thoroughly cleaned, and the mortality was also checked daily. 

### 2.2. Sampling for Growth Parameters, Proximate Composition, Histological Studies

The growth performance of the fish was evaluated in each treatment on the standard feeding basis of 8 weeks. Eight weeks is the international duration norm for fish growth trials, especially when evaluating the growth performance [43,45,46]. After the 56 days of the experiment, the survival percentage was recorded in each tank. On the 57th day, the fish were first starved for the whole day before sampling [43,46,47]. Then, on the 58th day, the fish were anesthetized with Tricaine methane-sulfonate MS-222, 60 mg∙L^−1^. After anesthesia, the first two parameters, final weight (g) and total length, were recorded for all fish. Among the 20 fish in each tank, 17 specimens were carefully dissected, and other parameters, such as liver weight and visceral weight, were also recorded. Moreover, from the 17 fish dissected, the gill, skin, dorsal muscle, whole intestine, foregut, midgut, and hindgut were sampled. Some were fixed in a solution of glutaraldehyde in a phosphate buffer (0.1 M, pH 7.0) for Hematoxylin & Eosin (H&E), and another sample was fixed in a formaldehyde solution and prepared for Scanning Electron Microscope (SEM) and Transmission Electron (TEM) microscope observation. For the dorsal muscle proximate composition analysis, a considerable amount, around 100 g, of the dorsal muscle was pulled from each of the 17 fish put together in plastic bags and immediately stored at −20 °C. The remaining three fish were not dissected but instead kept in the fridge at −20 °C, for the whole-body proximate composition analysis. 

### 2.3. Method for the Proximate Composition of the Whole-Body and Dorsal Muscle

Moisture, ash, crude proteins, and crude lipids were assessed following the method of the Association of Official Analytical Chemists [48]. Moisture was determined by drying ground samples in a forced-air oven at 105 °C for 24 h. Ash content was determined by incinerating samples at 600 °C for 24 h in a muffle furnace. Crude proteins were evaluated as Kjeldahl-nitrogen using a factor of 6.25, and crude lipids were analyzed by Soxhlet extraction with petroleum ether [49,50].

### 2.4. Histological Studies: H&E, SEM & TEM, Sample Preparation

The histological studies achieved in the present work include three different sample preparations. The first preparation is the H&E stain with light microscope observation. The second and third treatments were done for SEM observation and TEM observation, respectively. SEM gives us a 3D image with more details. The TEM observation was done to observe the liver cell’s inner structure for any histological difference or organelles pathology, focusing on the mitochondria structure shape, cristae arrangement, and the abundance and size of peroxisomes to identify if the long-term exposure to a lower pH could cause metabolic acidosis.

#### 2.4.1. Sample Preparation H&E (Hematoxylin and Eosin) Stain and Light Microscope Observation of Gills, Liver, Skin, Foregut, Midgut, and Hindgut

After the fish dissection, gill, liver, skin, foregut/duodenum, midgut/jejunum, and hindgut/ileum samples were collected. Each sample was fixed immediately after dissection in 10% formalin for 24 h. Later, in the laboratory, the samples were decalcified in 10% nitric acid, dehydrated in increasing alcohol concentrations, cleared in xylene, and impregnated and embedded in paraffin. Thin sample sections were stained with hematoxylin and eosin for histological description according to the standard method described by Munro [51] and used by Titford [52]. The gill, liver, skin, foregut midgut, and hindgut tissues were stained with H&E, put on small rectangle glass slides, and observed with the light microscope (Axiocam 506 color, ZEISS) [53,54,55]. All images were obtained in high-definition.

#### 2.4.2. Foregut, Midgut, and Hindgut Preparation for SEM Observations

For the first-step treatment called double fixation, the foregut was first fixed with 2.5% glutaraldehyde in a phosphate buffer (0.1 M, pH 7.0) for more than 4 h; then, it was washed three times in the phosphate buffer (0.1 M, pH 7.0) for 15 min at each step; then, it was post-fixed with 1% OsO4 in a phosphate buffer for 2 h and washed three times in a phosphate buffer (0.1 M, pH 7.0) for 15 min at each step. Then, the second step was a two-stage dehydration. In the first stage, it was dehydrated by a graded series of ethanol (30%, 50%, 70%, 80%, 90%, and 95%) for about 15 min at each step; then, it was dehydrated two times by alcohol for 20 min at each step, or stored in alcohol. In the second stage, the sample was dehydrated in a Hitachi Model HCP-2 critical point dryer. On the third and last step, the sample was coated with gold-palladium in a Hitachi Model E-1010 ion sputter for 5 min and observed in the Hitachi Model SU-8010 SEM. The image obtained here is a 3D image.

#### 2.4.3. Liver Sample Treatment and TEM Observation

After repeating the double fixation and the first stage dehydration used for SEM mentioned above, we did the infiltration process. Upon infiltration, the liver sample was placed in a 1:1 mixture of absolute acetone, and the final Spurr in a resin mixture for 1 h at room temperature, then transferred to a 1:3 mixture of absolute acetone; to the final resin mixture for 3 h, and finally to the Spurr resin mixture overnight. The sample was placed in Eppendorf-containing Spurr resin and heated at 70 °C for more than 9 h and was finally sectioned in a LEICA EM UC7 ultra-tome. Sections were stained by uranyl acetate and alkaline lead citrate for 5–10 min each and observed in a Hitachi Model H-7650 TEM. The Figure obtained here is a 2D view. 

### 2.5. Statistical Analysis

Statistical analyses were performed using the Software IMB SPSS Statistics 23.0.0.0. All data were tested for normality and homogeneity of variances by Kolmogorov–Smirnov and Levene’s tests, respectively. The data were subjected to a one-way analysis of variance (ANOVA), and a Tukey’s HSD test was used to compare significant differences between means at (*p* < 0.05) (*n* = 3 replicates). All the quantitative data are presented as mean ± SD (standard deviation).

## 3. Results

### 3.1. Seawater Physicochemical Parameters

The results of the measured seawater physicochemical parameters are presented in the Table 1.

### 3.2. Growth Parameters

The following Table 2 display the fish growth performance parameters after 8 weeks of growth trial, fed with the same diet.

Despite stocking juveniles with the same initial weight and feeding them with the same diet throughout the experimental period, the results revealed that the three treatments had varying growth parameters with respect to pH. Here, the data demonstrate that fish growth, fish weight, and fish length significantly decreased with decreasing pH. Regarding fish survival, there was no significant difference with *p >* 0.05 for all treatments. For the feed intake (FI) analysis, we observed that the fish in the Predict_A group had the highest values. However, while the feed intake increased from the Control to Predict groups, the decrease of the specific growth rate (SGR) was surprising. Statistically, there was no significant difference in the hepatosomatic index (HSI) or the condition factor (CF). Finally, considering the protein efficiency ratio (PER), a significant decrease was observed from the control group to the two predict groups. 

### 3.3. Proximate Composition of the Fish Samples

Table 3 shows the proximate composition of the whole body and the dorsal muscle. 

The Control group (pH of 8.10), with superscript “a”, shows significant differences compared to the Predict_B group (pH of 7.40), with superscript “b”. The Predict_A group, with superscript “ab”, is a middle value belonging to both the Control and Predict_B groups. Predict_A’s crude protein value explains the decrease, though it is not significantly different (*p* > 0.05) from either the Control or Predict_B groups.

Considering both the whole-body and dorsal muscle proximate composition, the moisture, crude lipid, and ash, having no superscript, indicate no significant difference between treatments. However, the crude protein content showed a decrease according to the lower pH. The decrease is significant between the Control group (pH of 8.10) and the Predict_B group (pH of 7.40). However, though the decrease is not significant from the Control to the Predict_A group, we can see from the superscript “ab” that this value can be classified to the significance of both the Control (not significant, superscript “a”) and the Predict_B group (significant, superscript “b”). 

### 3.4. SEM Observation of the Foregut Tissue

**Figure 2:**
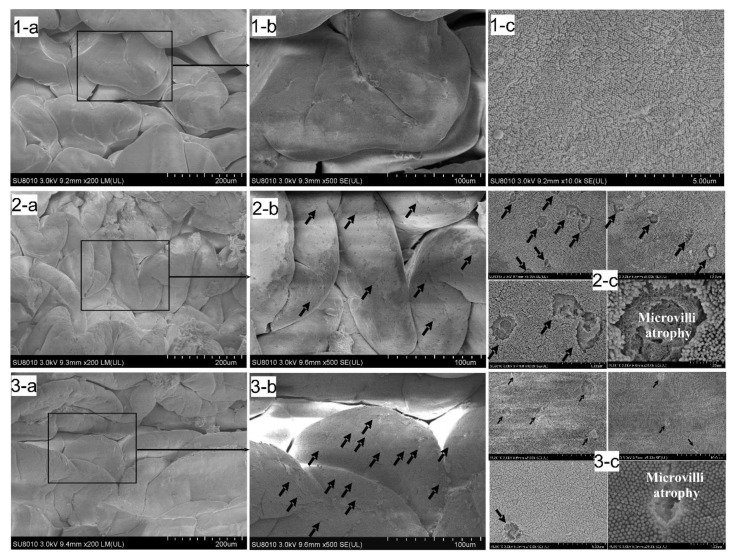
Scanning electron microscope observation of the foregut. (**1a**–**c**): Control Group, pH = 8.10 ± 0.01. (**2a**–**c**) Predict_A, pH = 7.80 ± 0.02, having a normal tissue distribution with multiple atrophies of the microvilli (black arrows). (**3a**–**c**) Predict_B, pH = 7.40 ± 0.02. Normal tissue distribution with pronounced histological atrophies. In this Figure (**2**), the black arrows pointing to the microvilli atrophy all along the brush border refer to the sawtooth-like structure observed in the next Figure (**3**), with red circles in pictures 2 and 3 and defined in picture 4.

Figure 2 is a 3D capture, obtained from SEM, and the Control group (1-a, 1-b, 1-c) displays a very regular tissue distribution, with almost no microvilli (air-like structure) atrophies over the brush border, suggesting that the level of seawater pH of 8.10 has a negligible effect on the intestine tissue. However, Predict_A (2-a, 2-b, 2-c) and Predict_B (3-a, 3-b, 3-c) showed multiple atrophies (black arrows) of the microvilli all along the brush border, signifying that seawater pH has a significant effect on the tissue. The Control group shows a regular structure of brush border without microvilli atrophy (1-c). In Predict_A (2-b, 2-c), and Predict_B (3-b, 3-c), the black arrows indicate multiple atrophies of the microvilli over the brush border.

### 3.5. H&E Stain: Observation of Foregut, Midgut, and Hindgut

**Figure 3:**
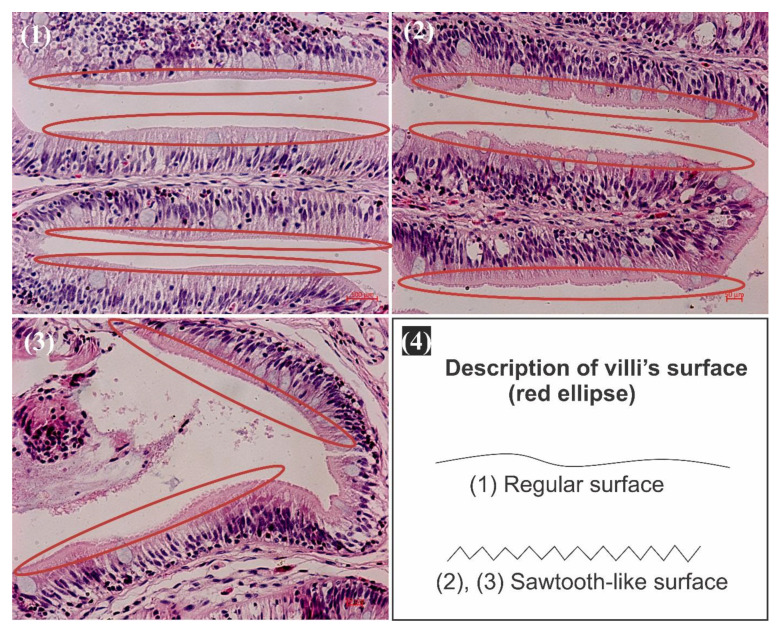
H&E stain and light microscope observation of the small intestine; duodenum villi and microvilli. 40 × magnification. (**1**) Control group with pH = 8.10; (**2**) Predict_A with pH = 7.80; (**3**) Predict_B with pH = 7.40. The red ellipse represents the brush border of the villi. (**4**) Description diagram of the villi surface.

**Figure 4:**
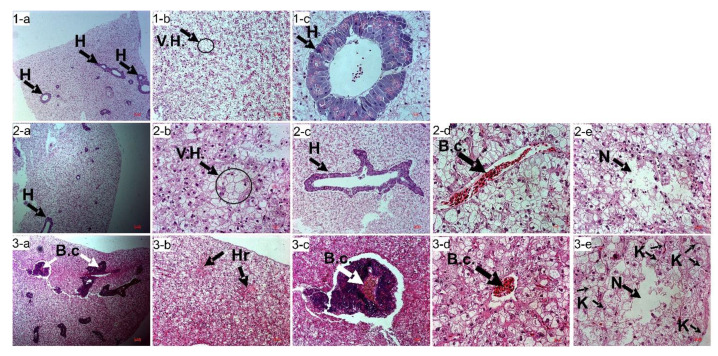
H&E stain of the liver tissue. Picture (**1a**–**c**) represents the Control group with a pH of 8.10 ± 0.01. Picture (**2a**–**e**) represents the Predict_A group with a pH of 7.80 ± 0.02. Picture (**3a**–**e**) represents the Predict_B group with a pH of 7.40 ± 0.02. B.c: Blood Congestion, H: Hepatopancreas; N: necrosis of hepatocytes; V.H: Vacuolation of hepatocytes; K: Kupffer cells; Hr: Hemorrhage.

In the Control group (Figure 3(1)), the villi’s brush border (red ellipse) has a regular plan surface, signifying a normal tissue distribution with almost no atrophies of the microvilli all along the surface. On the contrary, in the Predict_A and Predict_B groups (Figure 3(2),(3)), the surface of the villi is sawtooth-like, emphasizing the atrophy of the microvilli due to a lower pH or a higher acidification. A graphic of what we mean by regular surface and sawtooth-like surface has been presented (Figure 3 and Figure 4).

### 3.6. H&E Stain: Light Microscope Observation of the Liver 

In the Control group (Figure 4(1-a–c)) with normal seawater pH of 8.10 ± 0.01, a healthy liver’s characteristics are observed. The liver’s general structure is standard, with almost no blood congestion (B.c.) in all hepatopancreas (1-a, 1-c). There is no trace of cell necrosis in hepatocytes and no blood congestion inside the hepatopancreas. Moreover, Kupffer cells that could indicate infection are hardly found. In this Control group, only the hepatocytes’ vacuolation (V.H.) is seen in 1-b, in the black circle. In the Predict_A group with a pH of 7.80 ± 0.02, we also observe the hepatocytes’ vacuolation (V.H.) (2-b). Though we have no blood congestion in the hepatopancreas (2-c), we can identify blood congestion inside the blood vessel (2-d). At last, we identify the cell necrosis (2-e). Concerning the Predict_B group with a pH of 7.40 ± 0.02, we can observe a bloody liver with a hemorrhage emphasized with blood coloration (3-a to 3-e). It can be observed (3-a) that all hepatopancreas have blood congestion, magnified in (3-c). Two areas of hemorrhage (Hr) can be noticed (3-b). Blood congestion is also present in the central vein (3-d). The cell necrosis (N) can also be identified, with an emphasis on the abundance of Kupffer cells (K) (3-e).

### 3.7. H&E Stain: Light Microscope Observation of Skin

**Figure 5:**
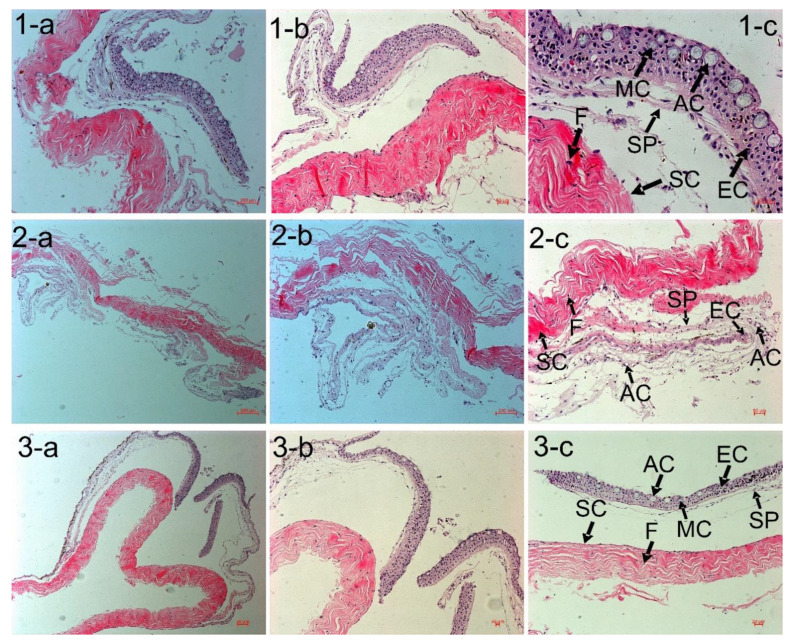
H&E stain of fish skin. (**1a**–**c**): Control group with seawater pH = 8.10 ± 0.01; (**2a**–**c**): Predict_A group with seawater pH = 7.80 ± 0.02; (**3a**–**c**): Predict_B group with seawater pH = 7.40 ± 0.02. Note that MC: Mucus, AC: Alarm cells, EC: Epithelial cells, F: Fibroblast represented in black spots in the stratum compactum structure, composed of stratum spongiosum (SP) and stratum compactum (SC) of the dermis.

In the three treatments, there was no histological atrophy related to the pH influence in the skin tissue (Figure 5). The main parts of the skin are identifiable. In the stratum spongiosum (SP) and stratum compactum (SC) of the dermis, we observed a normal skin with a well-defined stratified squamous epithelium with scattered mucous (MC), alarm cells (AC), epithelial cells (EC), and fibroblasts (F). Each of them has a standard structure distribution without any atrophies.

### 3.8. H&E Stain: Light Microscope Observation of the Gill

**Figure 6:**
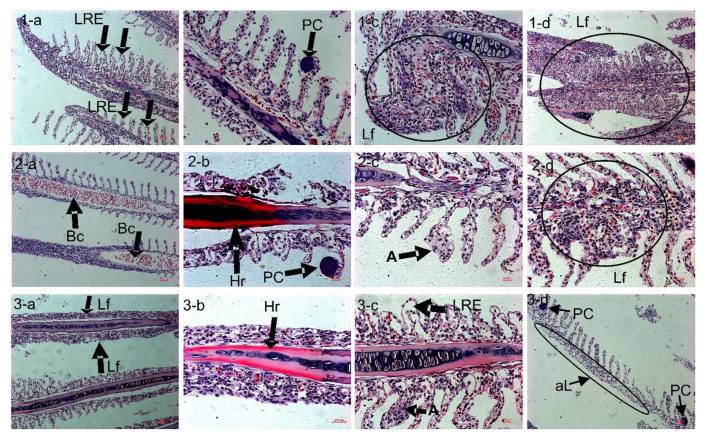
H&E stain and light microscope observation of gill tissue. (**1a**–**d**): Control group with seawater pH = 8.10 ± 0.01; (**2a**–**d**): Predict_A group with seawater pH = 7.80 ± 0.02; (**3a**–**d**): Predict_B group with seawater pH = 7.40 ± 0.02. A: Aneurysm; PC: Parasitic Cyst; aL: Absence of lamellae; B.c.: Blood Congestion; Lf: Lamellae fusion; Hr: Hemorrhage; LRE: Lifting of the Respiratory Epithelium (2 column fitting, color).

The phenomenon of hemorrhage in the gill’s filament appears in the Predict_A group (Figure 6(2-b)) and also in the Predict_B group (3-b). The blood congestion (B.c.) is observed at the inner structure of the lamellae in Predict_A (2-a). The lamellae inflammation called Aneurysm (A) is present in the Predict_A (2-c) and Predict_B groups (3-c). All three groups showed the presence of a parasitic cyst (PC) (1-b, 2-b, 3-d) and lamellae fusion (Lf) (1-c, 1-d, 2-d, 3-a). 

### 3.9. TEM Observation of Liver Cells (Inner Structure)

**Figure 7:**
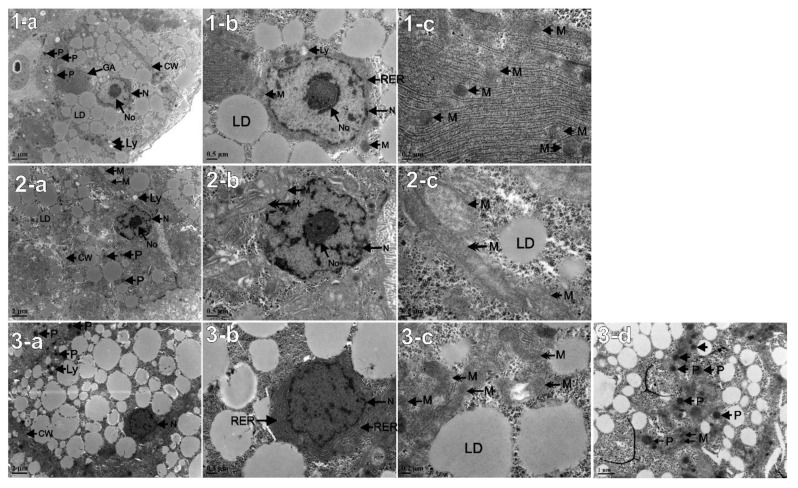
TEM observation of liver cells. (**1a**–**c**): Control group with seawater pH = 8.10 ± 0.01; (**2a**–**c**): Predict_A group with seawater pH = 7.80 ± 0.02; (**3a**–**d**): Predict_B group with seawater pH = 7.40 ± 0.02. No: Nucleolus, N: Nucleus; M: Mitochondrion; RER: Rough Endoplasmic Reticulum; GA: Golgi apparatus, CW: Cell Wall or Cell Membrane, Ly: Lysosome, P: Peroxisomes, LD: Lipid droplets (2 column fitting, color).

The first anomaly noticeable was a difference in the nucleus (N) shape (Figure 7(1-b) vs. Figure 7(2-b)/Figure 7(3-b)). The conventional round shape of the nucleus is observed in the Control group (1-b), while the Predict_A and Predict_B groups, on the contrary, present a crenated nucleus (2-b, 3-b). The absence of nucleolus is noticeable (3-b). Interestingly, Predict_B displays a greater abundance of peroxisomes (*p*) (3-c), which are hardly noticeable in the two other groups. Furthermore, a difference in the shape and size of the mitochondria is noticeable among the three groups; most of the mitochondria in the Control group have a conventional round shape, denoted with a single arrowhead (1-c). On the contrary, samples from Predict_A display some mitochondria with a very elongated structure, identified with a double head arrow (2-c). Furthermore, the samples from Predict_B also show mitochondria with an elongated and angular shape, also indicated by a double head arrow (3-d).

## 4. Discussion

Our results illustrate the significant negative effect of OA on the health, growth, and meat quality of the black sea bream (*Acanthopagrus schlegelii)* from the end of the 21st century. If we consider the single effect of ocean acidification, the fish are susceptible to survive up to that period. However, as the phenomenon of acidification is always coupled with warming, it is reported that a threat to the survival of black sea bream is expected, as well as to the growth health and the meat quality of this species by the end of 2100 and beyond [20]. 

### 4.1. Water Physicochemical Parameters 

The projected scenario represents a very significant decrease in pH, as can be seen by our measured pH. Consequently, there will be a significant reduction of the concentration of carbonate ions in the water [CO_3_^2−^], and also a significant decrease of the state of aragonite and calcite (Ωara and Ωcal). The more significant the decrease in the concentration of carbonate ions [CO_3_^2−^], the more significant the increase in the concentration of dissolved inorganic carbon in the seawater.

In case the projected ocean pH values effectively reach 7.80 and 7.40 by 2100 and beyond, as projected by IPCC [1], the present study has provided in Table 1 an overview of the expected seawater physicochemical parameters. From the measured parameters (S, T°, pH, TA), we were able to calculate others (pCO_2_, HCO_3_^−^, CO_3_^2−^, DIC, Ωara, Ωcal) using the software CO2cal 1.2.0. From the equation pH =-log [H^+^] [12,36], the acidity concentration can be deduced as [H^+^] = 10^(−pH)^. This means that the projected pH values 8.10, 7.80, and 7.40 give acidity values of 7.94 × 10^−9^, 15.85 × 10^−9^, and 39.81 × 10^−9^ in Control, Predict_A, and Predict_B, respectively. Compared to the Control group, Predict_A and Predict_B represent a 99.53% and a 401.19% increase in acidity, respectively, which is highly significant.

### 4.2. Proximate Composition Showing a Reduction in Meat Quality

Except for crude proteins, there was no significant difference in moisture, crude lipids, and ash content among the treatments (Table 3). For both the whole body and the dorsal muscle, a significant decrease was observed in the crude protein content from the Control to the Predict_B groups. This significant decrease implies a reduction in the meat’s nutritional quality. Nevertheless, the decrease was not significant from the Control to the Predict_A groups, but we can see from the superscript “ab” that this value can be classified according to the significance levels of both the Control group (not significant) and the Predict_B group (significant). Another parameter is therefore necessary to confirm which conclusion should be considered. The PER values in the growth parameters shown in Table 2 confirmed this reduction in meat quality, because we see the same significant decrease of protein content from the Control to the Predict_B groups. However, in contrast to the case of crude proteins, the PER displayed a significant decrease from the Control to Predict_A groups. The best approach to explain this slight difference is to calculate the PER and evaluate the crude protein content. There formula used to calculate the PER—PER = Weight Gain (g)/Protein intake on a dry basis (g)—is very precise. The crude protein content is evaluated through chemistry titration, following the international reference Kjeldahl method [56], which is not as precise as the PER value calculation. It is therefore possible that during the three replicates evaluation of the crude protein content, the resulting standard deviation was larger and, consequently, the significance decreased. At least the decreasing trend is observed in both the PER and the crude protein, which are both significant in the Predict_B group. When scientifically studying the risks, it is always better to consider the worst case. Consequently, there may be the risk of a significant decrease in the meat quality of black sea bream from the end of the 21st century. Studying the impact of OA on mussels, Martin et al. [57] reported a negative effect on meat quality. Furthermore, the studies of Rossoll et al. [58] and Jin et al. [59] also reported a decrease in fish samples’ meat quality due to OA. A possible reason is that the atrophy of the microvilli in the small intestine will reduce the amount of nutrients absorbed. This explains why, in the Predict_A and Predict_B groups, the microvilli atrophy in the duodenum jejunum and ileum decreases the nutrient absorption, affecting the crude protein gain and resulting in slow growth, lower weight gained, lower total length, and, consequently, a reduction in fish meat quality.

### 4.3. Growth Parameters Revealing a Significant Low Growth and Confirming the Reduction in Meat Quality

There was no significant difference in the Hepatosomatic index (HSI), and the Condition factor (CF) indicated the same feeding intensity. Although the fish in all the treatments were fed with the same feeding intensity, the data revealed a low growth rate in Predict_A and Predict_B, despite the higher feed intake (FI) in these Predict groups than in the Control group. In simple terms, the fish ate more than normal but still grew lesser than they should, which was observed earlier by Zhang et al. [60] while studying subtidal scavengers *(Nassarius conoidalis)*. The growth parameter data in Table 2, which include the final length (FL), the weight gained (WG), and the specific growth rate (SGR), show a significant decrease (*p* < 0.05). This indicates that a decrease in seawater pH led to a significant decrease in fish growth—the more the seawater decreases the more the fish growth is affected. The significant decrease of the feed efficiency (FE) and the protein efficiency ratio (PER) for the Control group to the Predict_A and Predict_B groups indicate that the feed consumed is less efficient in the two prediction groups than in the Control group. The possible reason may be the lower pH, causing an atrophy of the microvilli that will lead to less nutrient absorption in the small intestine. Inversely to the significant decrease of FE, we see a significant increase in the feed conversion ratio (FCR) in the Control group vs. the Predict_A and Predict_B groups, because the formula of the FE is inversely proportional to that of the FCR (see the FE and FCR formula under Table 2). The FCR is defined as the amount of feed necessary to produce a unit of animal weight. This indicates that more feed is necessary to produce a unit of animal weight in the Predict_A and Predict_B groups than in the Control group. This latter statement can also be explained by the loss of nutrients through the small intestine. Research studies on *Sparus aurata* and *Nassarius conoidalis* published by Araujo et al. [20] and Zhang et al. [60], respectively, highlighted that OA decreased fish survival, boosted the energy demand, and reduced the physiology and scope for growth. 

### 4.4. The Survival Rate and the Histology of Liver and Gill Tissue Showing Degrading Effects on Fish Health

The results on survival and the histological studies of liver and gills tissues have revealed a considerable impact on fish health. The survival rate (SR) had no significant difference (*p* > 0.05) among treatments, suggesting that considering the only factor of pH decrease in the 21st and 23rd centuries, the fish may be able to survive. Nevertheless, it has been reported that if we add temperature as a second factor, the fish survival will be affected from the 21st century and well beyond [20]. Concerning the fish health evaluation, we noticed the damage of the fish liver in Predict_A and much more in the Predict_B group. Regarding the liver tissue observation after the H&E stain shown (Figure 4), the light microscope revealed necrosis, blood congestion, vacuolation of the hepatocytes, hemorrhage, and an increased number of Kupffer cells on Predict_A and Predict_B. The anomalies mentioned above are signs of liver damage, leading to the perturbation of the fish liver’s optimal function [17,61].

Gill histology showed a slight difference among treatments where signs of blood congestion were observed in the Predict_A (2-b, 2-d) and Predict_B groups (3-d) shown in Figure 6. Except for the blood congestion, each of the three groups showed the same anomalies: the absence of lamellae, lamellae fusion, aneurysm, and parasitic cyst. Therefore, they cannot be considered an effect of low pH [62,63,64].

### 4.5. Histology Showing No Atrophy on Fish Skin Tissue 

Interestingly, no observable effect of acidification was noticed on the fish skin (Figure 5). This suggests that OA’s future prediction at the end of the 21st century and beyond will have minimal or no effect on black sea bream skin. This is possible because the ocean pH would still be within the acceptable range for fish skin to endure [65,66]. 

### 4.6. TEM Observation of Fish Liver Cell’s Inner Structure, Revealing Signs of Metabolic Acidosis

The TEM observation revealed an elongated and angular shape of mitochondria in the Predict_B group, a condition known as lactic acidosis manifestation [67]. Lactic acidosis is a form of metabolic acidosis. The abundance of peroxisomes was also noticed in this 3rd group, with the highest acidification value [67]. This result suggests that through fish respiration, when the blood gains oxygen, CO_2_ also gets into the blood through the gill and comes into direct contact with the acidic seawater [36,68,69]. This result suggests that when the blood gains dissolved oxygen during respiration, the dissolved CO_2_ also gets into the blood through the gill because of its direct contact with the acidic seawater [36,68,69]. Not being able to regulate the excess of dissolved CO_2_ in the blood, the fish will experience a decrease in the blood pH value, causing acidosis. This inefficiency of the acid–base regulation is due to the long-term exposure to the acidic environment. [70] demonstrated earlier that a value of pCO_2_ = 1000 µatm could lead to metabolic acidosis. Since the present study recorded a high pCO_2_ value of 1993.71 ± 102.12 µatm in Predict_B, metabolic acidosis was expected.

### 4.7. Histological Studies of Small Intestine Revealing Microvilli Atrophy

The small intestine’s wall contains many different anatomical structures, functioning either to provide a defense for the small intestine tissues or to absorb nutrients from food. The microvilli are the small, hair-like projections that extend outward from the small intestine wall [71], whose function is to absorb nutrients from food [72]. Our results showed that both the light microscope and the SEM observation of samples of the small intestine in the Control, Predict_A, and Predict_B groups (Figure 2, Figure 3) showed the same results. The 3D pictures (Figure 2) provide details of (Figure 3) the black arrows shown (Figure 2)**,** obtained after SEM, and explain that the sawtooth-like structure observed (Figure 3) is actually due to multiple atrophies of the microvilli throughout the brush border of the duodenum. These atrophies suggest that the number of microvilli responsible for absorption is significantly reduced, resulting in nutrient loss. Though the image presented (Figure 2 and Figure 3) is just the small intestine’s foregut, the analysis and observation were also conducted on the midgut and hindgut. Similar results regarding the atrophy of the microvilli were obtained. To the best of our knowledge, no previous study has reported atrophies of the microvilli on the small intestine under the effect of OA, thus making our result a novel discovery. However, a broader look revealed that the microvilli atrophy observed (Figure 2 and Figure 3) is similar to that of a well-known human disease called microvilli inclusion disease (MVID). In his publication, Sidhaye, et al. [73] discovered that zebrafish exhibited cellular attributes of human microvillus inclusion disease. The MVID investigated by researchers revealed microvilli atrophy, metabolic acidosis, and diarrhea [71,74,75,76,77,78,79,80,81]. Likewise, in our experiment, samples from the Predict_A and Predict_B groups revealed the same anomalies of microvilli atrophy, signs of metabolic acidosis, and nutrient loss. This nutrient loss can be considered as “diarrhea” in humans. [78] stated that many patients with MVID experience liver dysfunction, supporting our discussion in Section 4.4. 

## 5. Conclusions

This research found that pH values of 7.80 at the end of the 21st century and 7.40 in the 23rd century would significantly decrease the crude protein content of black sea bream and the protein efficiency ratio. Under the projected OA pH, the growth performance data revealed that black sea bream eat more than normal but grow less than normal. Histological studies revealed that OA had caused multiple atrophies on small intestine microvilli, causing a significant decrease in nutrient absorption, resulting in a lower weight gain and a lower specific growth rate. The observation of samples on a scanning electron microscope, a transmission electron microscope, and an optical microscope demonstrated liver and gill anomalies, suggesting a negative effect on the health of black sea bream. Given that black sea bream is one of the most resistant fish to disease and environmental change, it can be deduced that much more varieties of fish—such as yellowtail (*Seriola quinqueradiata*), flounder (*Paralichthys olivaceus*), purplish amberjack (*Seriola purpurascens*), red sea bream (*Pagrus major*), puffer (*Takifugu rubripes*), striped jack (*Pseudocaranx dentex*), sea perch (*Lateolabrax* spp.), goldstriped amberjack (*Seriola aureovittata*), Schlegel’s black rockfish (*Sebastes schlegelii*), striped beak perch (*Oplegnathus fasciatus*), or dark-banded rockfish (*Sebastes inermis*), just to cite these, all having a lower resistance—may be affected [41]. Consequently, it can be presumed that if mitigation measures are not taken to curb the emission of CO_2_ in the atmosphere, OA is expected to cause a significant degrading effect on fish health, fish growth, and fish meat quality. Fish survival could also be threatened beyond 2100 if we consider the effect of both pH and temperature. The impact on fish health and, possibly, on the survival rate will affect the fish population, leading to a decline in marine resources. Given the likely risks of future OA in black sea bream, and the even greater risks in more sensitive species, CO_2_ mitigation is needed. For example, we highly recommend and encourage the use of green energy, via electric vehicles or the planting of trees, especially in the most polluted environments, to absorb the excess CO_2_ released into the atmosphere. Furthermore, this study helps aquaculture practices by demonstrating the implications of low pH on fish health, survival, meat quality, and feeding efficiency. Further analyses are being carried out on this same experiment in the current year (2021), one on brain transcriptomics and another on fish behavior assessment by computer vision.

## Figures and Tables

**Table 1:** Seawater parameters during the experiment.

	Control	Predict_A	Predict_B
Targeted pH	8.10	7.80	7.40
Seawater parameters			
Salinity (g∙L^−1^)	27.13 ± 1.06	27.10 ± 1.10	27.17 ± 1.09
Temperature (°C)	27.53 ± 0.85	27.51 ± 0.74	27.47 ± 0.87
Measured pH	8.10 ± 0.01 ^a^	7.80 ± 0.02 ^b^	7.40 ± 0.02 ^c^
TA	2051.43 ± 16.11 ^b^	2097.40 ± 20.15 ^a^	2065.39 ± 17.22 ^ab^
pCO_2_ (µatm)	321.37 ± 11.48 ^c^	749.12 ± 27.03 ^b^	1993.71 ± 102.12 ^a^
HCO_3_^−^ (µmol∙kg^−1^)	1573.43 ± 20.10 ^c^	1823.60 ± 14.65 ^b^	1947.91 ± 18.77 ^a^
CO_3_^2−^ (µmol∙kg^−1^)	196.82 ± 1.45 ^a^	113.41 ± 4.53 ^b^	48.57 ± 1.93 ^c^
DIC (µmol∙kg^−1^)	1779.15 ± 19.01 ^c^	1957.74 ± 16.45 ^b^	2054.48 ± 20.80 ^a^
Ωara	3.29 ± 0.024 ^a^	1.90 ± 0.08 ^b^	0.81 ± 0.03 ^c^
Ωcal	5.07 ± 0.04 ^a^	2.92 ± 0.12 ^b^	1.25 ± 0.50 ^c^

Values are presented as mean ± SD (standard deviation), and *n* = 3 replicates values with different superscripts in the same row differ significantly with (*p* < 0.05). The partial pressure of CO_2_, pCO_2_ (µatm), bicarbonate ions (HCO_3_^−^) (µmol∙kg^−1^), carbonate ions (CO_3_^2−^) (µmol∙kg^−1^), dissolved inorganic carbon (DIC) (µmol∙kg^−1^), and the saturation state of aragonite and calcite, respectively Ωara and Ωcal, were all calculated from the measured salinity, measured pH, measured temperature, and measured total alkalinity TA, at an atmospheric pressure of 1atm = 1013.25 kilobar, using the open-source software “CO2cal 1.2.0” (the updated version of CO2SYS).

**Table 2:** Growth parameters of black sea bream (*Acanthopagrus schlegelii)* after 8-week growth trial.

	Control	Predict_A	Predict_B
Targeted pH	8.10	7.80	7.40
Initial weight (IW) (g/fish)	2.73 ± 0.01	2.72 ± 0.02	2.73 ± 0.01
Final weight (FW) (g/fish)	20.86 ± 0.25 ^a^	17.54 ± 0.49 ^b^	15.80 ± 0.06 ^c^
SR ^1^ (%)	100 ± 0.00	100 ± 0.00	98.33 ± 2.89
WG ^2^ (%)	664.01 ± 6.79 ^a^	545.80 ± 21.57 ^b^	479.43 ± 3.73 ^c^
SGR ^3^ (%/day)	4.07 ± 0.02 ^a^	3.73 ± 0.07 ^b^	3.51 ± 0.01 ^c^
FI ^4^ (%/day)	29.38 ± 0.32 ^b^	30.81 ± 0.66 ^a^	30.39 ± 0.21 ^ab^
FCR ^5^	1.04 ± 0.01 ^b^	1.14 ± 0.04 ^a^	1.17 ± 0.01 ^a^
FE ^6^ (%)	95.84 ± 0.88 ^a^	87.45 ± 3.21 ^b^	85.17 ± 0.99 ^b^
HIS ^7^ (%)	2.07 ± 0.24	1.91 ± 0.24	1.90 ± 0.23
CF ^8^ (g/cm^3^)	2.47 ± 0.09	2.93 ± 0.23	2.76 ± 0.22
PER ^9^	2.44 ± 0.02 ^a^	2.23 ± 0.08 ^b^	2.17 ± 0.03 ^b^

Values are presented as mean ± SD (Standard deviation) (*n* = 3); values with different superscripts in the same row differ significantly with (*p* < 0.05). ^1^ SR (Survival rate) (%) = 100 × (Final fish number/Initial fish number). ^2^ WG (Weight gain) (%) = 100 × (Final body weight–Initial body weight)/Initial body weight. ^3^ SGR (Specific growth rate) (%/day) = 100[ln (Final body weight)–ln (Initial body weight)]/Time(day). ^4^ FI (Feed intake) (%/day) = [100 × Dry diet fed (g)/(Final body weight (g) + Initial body weight(g))/2]/Feeding duration (in one day). ^5^ FCR (Feed Conversion Ratio) = the amount of feed necessary to produce a unit of animal weight increase = dry diet–fed (g)/wet weight gain (g). ^6^ FE (Feed efficiency) (%) = 100 × Fish body weight (g)/Dry feed(g) ^7^ HSI (Hepatosomatic) (%) = 100 × Liver weight (g)/Body weight (g). ^8^ CF (Condition factor) (g/cm3) = 100 × (Liver weight/(Body length(cm))3. ^9^ PER (Protein efficiency ratio) = Weight Gain (g)/Protein intake on a dry basis (g).

**Table 3:** Proximate composition of the whole-body and dorsal muscle.

	Control	Predict_A	Predict_B
Proximate composition (%)	8.10	7.80	7.40
Whole-body			
Moisture	71.08 ± 0.18	70.48 ± 0.76	70.96 ± 0.69
Crude Protein	52.86 ± 1.08 ^a^	50.43 ± 1.20 ^ab^	48.88 ± 1.16 ^b^
Crude Lipid	20.71 ± 0.71	21.38 ± 0.53	20.35 ± 0.60
Ash	5.02 ± 0.05	5.08 ± 0.18	5.08 ± 0.17
Dorsal Muscle			
Moisture	76.39 ± 0.42	76.58 ± 0.35	76.57 ± 0.10
Crude Protein	70.98 ± 1.12 ^a^	69.61 ± 1.52 ^ab^	67.87 ± 0.87 ^b^
Crude Lipid	6.06 ± 1.08	5.56 ± 0.57	5.63 ± 0.20
Ash	4.94 ± 0.08	5.24 ± 0.53	5.03 ± 0.23

Values are presented as mean ± SD (standard deviation) (*n* = 3 replications); values with different superscripts in the same row differ significantly (*p* < 0.05).

## Data Availability

The data presented in this study are available on request from the first author Fabrice Arnaud Tegomo by contacting 11734055@zju.edu.cn.
